# Medawar and the immunological paradox of pregnancy: in context

**DOI:** 10.1093/oxfimm/iqaa006

**Published:** 2020-12-14

**Authors:** Victoria Male

**Affiliations:** Department of Metabolism, Digestion and Reproduction, Imperial College London

## Abstract

In 1953, Peter Medawar defined ‘the immunological paradox of pregnancy’, whereby the semi-allogeneic foetus can survive for 9 months in its mother, while a semi-allogeneic graft would be rejected. Here, I revisit the immunological paradox of pregnancy, setting it in the context of the time in which it was proposed. I go on to examine the extent to which Medawar’s ideas on the subject have stood the test of time and how they have shaped reproductive immunology.

In this inaugural issue of Oxford Open Immunology, it seems fitting to consider the legacy of Oxford’s most famous immunologist and the founder of the modern discipline: Peter Medawar. This is not, of course, to diminish the achievements of those who came before him: Pasteur, Metchnikoff and Ehrlich arguably each has a claim to the title ‘the first immunologist’. Nor is it to detract from the contributions of those others working at the same time who also elucidated the mechanisms of tolerance. Medawar himself was generous in sharing the credit (and indeed the prize money) for these discoveries [1].

Nonetheless, Medawar merits special consideration because of his immense influence on our thinking as immunologists. Partly, this is because of the significance of his discoveries, but his many personal qualities have also shaped his legend. He was an adept scientific communicator, ‘perhaps the greatest scientific writer of his generation’ [2]. He also took his role as a mentor to younger scientists very seriously and was unusually forward-thinking in his views on the contributions to science that could be made by members of underrepresented minorities [3].

## TRANSPLANTS AND TOLERANCE

Tellings of Medawar’s story often begin on the sunny Sunday afternoon in 1940, when a British bomber plane crashed into a back garden near Medawar’s Oxford home. Although the pilot survived, he suffered severe burns. Medawar, already involved in research on antibiotics, was called to the war wounds hospital to look at the pilot. This proved a turning point for him, spurring him to work tirelessly to overcome the problem of graft rejection, which prevented skin from unrelated individuals from being used to cover the extensive burns sustained by the injured airman, and others like him [4].

Working with surgeon Tom Gibson, Medawar began by undertaking the first systematic study of the process of graft rejection [5]. Their patient was a 22-year-old woman who had fallen against a gas fire and sustained burns covering the entire upper right of her body. To cover the area, the pair transplanted a patchwork of ‘pinch’ grafts, some of which were taken from the patient herself and some of which came from her brother. Later, a second set of grafts were transplanted from the brother. By taking biopsies and examining the grafts histologically, Medawar found that at first both sets of grafts healed and became vascularized, but a few days later, the grafts from the patient’s brother became infiltrated with immune cells and were rejected shortly after. The second set of grafts from her brother underwent immune cell infiltration and rejection more rapidly, a hallmark of the immune response.

To investigate this in more detail, Medawar turned to animal models. Working in rabbits, he showed that rejection was preceded by lymphocyte infiltration. Furthermore, second grafts were consistently rejected faster than first grafts, if they came from the same donor, but second grafts from an unrelated donor were rejected at the same rate as first grafts [6, 7]. These experiments established beyond doubt that graft rejection was an immune phenomenon and that some specific antigen, that differed between individuals, was the cause of the rejection.

Working now with Rupert Billingham and Leslie Brent, Medawar was inspired by work from Ray Owen, which came to his attention via Macfarlane Burnet. Owen had found that twin calves shared a blood circulation via the placenta *in utero* and that cells from each calf could continue to exist in the other [8]. This made sense of an observation from Medawar’s team, that twin calves could invariably accept skin grafts from each other, even when they were not identical [9] and led Medawar to test whether experimentally-induced chimerism could produce tolerance in a similar way [10]. Using inbred mice, in which the genetics of transplantation antigens were already beginning to be understood as a result of the work of George Snell and Peter Gorer, they injected a litter of CBA mice *in utero* with tissues from strain A mice. When the chimeric pups were 8 weeks old, they were grafted with strain A skin. In contrast to normal CBA mice, the chimeric mice tolerated the strain A grafts. Finally, Medawar had solved the transplantation problem, at least theoretically, and in 1960 he shared the Nobel Prize with Macfarlane Burnet for this work.

Although his Nobel Prize-winning work held out the hope of a solution to the transplant problem, Medawar recognized that the generation of tolerance in this way was never going to be practical [11]. Instead, he pointed to its ‘moral’ significance, putting ‘new heart into the many biologists and surgeons who were working to make it possible to graft, for example, kidneys from one person to another’ [4]. In their later work, Medawar and his team went on to identify ways in which the immune system could be suppressed to prolong the life of grafts and they were among the scientists to demonstrate the utility of corticosteroids [12] and lymphocyte-depleting antibodies [13, 14] in immunosuppression for transplantation, approaches which are still in clinical use today.

But even on home ground, Medawar was not infallible, as he himself was first to admit [3]. ‘We shall come to regard the presence of lymphocytes in the thymus’, he wrote, ‘as an evolutionary accident of no great significance.’ [15] We would do well to remember this. Yet his ideas, even when mere conjecture, have come to have disproportionate influence in reproductive immunology.

## THE PARADOX OF PREGNANCY

When I talk about transplantation immunology, I cast Medawar as a visionary and a hero. Yet I caution my graduate students, who are reproductive immunologists, against beginning every piece of writing with a discussion of Medawar’s 1953 lecture on the immunological paradox of pregnancy [16]. To do so has become somewhat of a knee-jerk response, but I do not counsel them against it simply because it is a cliché. Rather, I contend that to engage with Medawar on his own terms is to consider the foetus as an organ graft. This is inappropriate for two reasons. First, as Medawar himself noted, the placenta forms an anatomical barrier between the mother and foetus, so their circulations do not mix [16]. Second, when a pregnancy fails we do not see an influx of T cells, so failure of pregnancy is very unlike that of an organ graft [17].

In his 1953 lecture, Medawar proposed three ways in which the foetus could avoid recognition by the maternal immune system. First, the placenta forms an anatomical barrier. Medawar suggested that this is likely to be ‘by far the most important’ factor in preventing allorecognition of the foetus [16], pointing out in an article written at around the same time that the ‘vascular quarantine’ of the foetus has some parallels with the immunological privilege of the cornea [11]. With the benefit of a further seven decades of research, we can agree with him. Indeed, we now understand that the immunology of the placenta is sophisticated and unique. The main placental cells in contact with the maternal blood are villous trophoblast cells, which cover the placental villi and act as the site of exchange between the mother and foetus. These cells do not express any MHC molecules at all, effectively making them immunologically invisible. The outer layer of these cells has a thick glycocalyx [18], further impairing immune recognition, as well as forming a syncytium, which is likely to improve their ability to resist cytolytic attack. The cells of the placenta that invade into the lining of the uterus, extravillous trophoblast, are still more immunologically interesting. These express a unique combination of MHC molecules (HLA-C, HLA-E and HLA-G [19–21]), which have limited ability to be recognized by T cells, but are major ligands for NK cells and macrophages. These innate immune cells form the majority of the immune population in the lining of the uterus and the observation that extravillous trophoblast seems to be calibrated to activate innate immune cells points to an important role for this interaction in the success of pregnancy.

The other two ways in which Medawar suggested the foetus could avoid immune recognition have turned out to be less reflective of the facts as we now understand them. He suggested that early in pregnancy, the antigenic immaturity of the foetus protects it from allorecognition but later, as the foetus begins to express antigens, generalized immunosuppression of the mother, mediated by increased cortisol, allows the foetus to persist. We now know that the foetus itself expresses MHC molecules from before 8 weeks of gestation [22], with expression of MHC by extravillous trophoblast cells occurring earlier than 6 weeks [20]. And although the immune system is somewhat altered during pregnancy, generalized immunosuppression is neither a feature of pregnancy, nor required for a successful outcome [23, 24]. Indeed, the outcomes of pregnancy are worse in women who are genuinely immunosuppressed [25].

But Medawar’s arguments can be better understood if they are taken in the context of the time in which they were made. The cause of haemolytic disease of the newborn, in which maternal anti-Rhesus D antibodies cross the placenta and attack foetal red blood cells, had recently been described [26]. This disease often manifests in a second pregnancy following sensitization in the first and this striking parallel with his own observations on the more rapid rejection of second grafts seems to be one of the things that drew Medawar’s attention to the subject. The observation that haemolytic disease of the newborn presents in the second or third trimester gave rise to Medawar’s suggestion that in the first trimester the foetus is immunologically inert. Further, the recent discovery of corticosteroids as a method of improving graft survival in rabbits [12] led him to speculate that they could act similarly in pregnancy.

Naturally, Medawar viewed pregnancy through the lens of transplantation and it is tempting for those of us who stand on his shoulders to do likewise, despite his repeatedly cautioning us that his thoughts on the matter might not stand the test of experiment. But to do so is to make two mistakes.

First, to consider the foetus as an allograft is to default to thinking of maternal immune cells as infiltrating from the circulation, when we now understand that immune cells in tissues develop and behave rather differently from those in the blood [27–29]. Using the blood as our default comparator can therefore mislead us into believing that we have discovered aspects of the uterine environment that promote tolerance to the foetus, when we are in fact observing a feature of the immune system that applies to tissues in general. For example, Tregs are enriched in the lining of the uterus [17], but this is equally true of tissues such as the gut and liver [30, 31]. When either total T cells, or T cells depleted of Tregs, are transferred into pregnant mice, we see increased foetal resorption in the absence of Tregs, and this affects allogeneic more than syngeneic pregnancies [32]. Yet equivalent experiments carried out in colitis models also demonstrated pathology in the absence of Tregs [33]. Indeed, the absence of Tregs causes loss of immune homeostasis in a number of organs [34], so these cells, important though they are for tolerance to the conceptus, are clearly essential for tolerance in general. Similarly, the uterine environment is characterized by high expression of the anti-inflammatory cytokine TGFβ [17], but this is equally true of other organs, such as the gut and liver [30, 31].

Secondly, this adaptive immune-centric view can distract us from the role of innate immune cells. In 1953, Medawar could not have imagined the complexity and importance of the innate immune system that would be discovered in the coming decades. When, sometime later, attention turned to innate immune cells in the uterus, their sheer volume in the first trimester suggested they were important for implantation. Uterine NK cells produce factors that attract extravillous trophoblast, promote angiogenesis and act on macrophages to promote tissue remodelling [35–38]. Immunogenetic studies have shown that pregnancies in which the mother’s NK cells can be activated by foetal HLA-C molecules on extravillous trophoblast are more likely to be successful [39–41], indicating that this is a key interaction promoting placentation. HLA-G appears to have a role in stimulating cytokine production by macrophages in the lining of the uterus, which also shapes the implantation site [42]. In addition to promoting placentation, innate immune cells in the lining of the uterus also provide defence against vertical transmission of infection [43, 44], itself important for reproductive success given the relative immunological vulnerability of the foetus.

## OF MICROBES AND MALIGNANCY

Although the immune response elicited by the conceptus at the site of implantation is unlike that elicited by a graft, Medawar noted that mice immunized with embryonic cells at another site can produce an immune response to them. This provides some protection against chemically-induced tumours, a phenomenon that results from the re-expression of foetal antigens by tumour cells. [45]. Medawar’s interest in this was as a route by which it might be possible to produce anti-tumour immunity, but this line of thinking also highlights some parallels between placental and tumour cells. As well as sharing the expression of certain carcino-embryonic antigens, both are extremely proliferative and invasive, with trophoblast invasiveness closely correlating with susceptibility to carcinomas between species [46]. A number of molecular mechanisms are common to trophoblast and malignant cell invasion [47], but a key difference is that in the case of trophoblast, this is tightly controlled by cells in the lining of the uterus, including stromal [48] and immune [49] cells. Clearly, trophoblast has the potential to behave like a malignant cell, since when the conceptus implants ectopically or on a scar from a previous caesarean section, specialist immune and stromal cells are absent and invasion is uncontrolled.

In his later years, Medawar became interested in harnessing the immune response to tumours, yet he never became engaged in the role of the immune system in responding to pathogens. On the subject of antigenic variation between individuals, he professed himself to be ‘under some obligation to rack [his] brains for evidence of any good it might conceivably do’ [16]. Writing some quarter of a century before the discovery of MHC-restricted antigen recognition, he can be forgiven for not intuiting that pathogens impose selection on the MHC, driving increased polymorphism [50], but his failure to consider infectious disease as a factor that might account for this aspect of the immune response is nonetheless telling. Yet it left a fertile niche for those who came after him to unravel the mechanisms by which micro-organisms raise immune responses when purified antigens do not and, conversely, why in some cases even micro-organisms may be tolerated.

A major anatomical site at which this occurs is the gut. One estimate suggests there are more lymphocytes in the gut than in all the secondary lymphoid tissue combined [31], yet in healthy individuals, this titanic force is kept in check in the face of continuous challenge by microbial and food antigens. In his Nobel Lecture, Medawar nodded to the ability of antigens introduced orally to induce tolerance [1], a property that is now thought to be at least partially the result of the tolerogenic environment both required and induced by the microbiota [31].

There are a number of parallels between the conceptus and the microbiota. Both are allowed to survive because the relationship also benefits the host. In the case of the conceptus, it allows the mother to pass on her genes, while the relationship with the microbiota allows access to otherwise inaccessible nutrients [51]. In both cases, the immune system must maintain tolerance to the foreigner, while limiting its potential for invasion. The mechanisms by which tolerance is achieved share points in common, such as an enrichment for unconventional lymphoid cells, regulatory T cells and a reliance on TGFβ [17, 31]. On the contrary, the mechanisms that limit placental invasion rely on stromal and innate immune cells in the lining of the uterus [48, 49], whereas in the gut commensal microbes are controlled by soluble antimicrobial factors and antibodies in the mucus [31]. These parallels may lead us to contemplate how the development of reproductive immunology might have differed if it had arisen not from Medawar’s idea of the foetus as an allograft, but rather as a species of mucosal immunology.

## THE MAN, THE MYTH

This piece was originally conceived as a discussion of Medawar’s influence on our thinking as immunologists, and I had intended to argue that it has not been universally helpful. Particularly in reproductive immunology, the way that we use Medawar’s 1953 lecture on the immunological paradox of pregnancy has often led us astray. And yet, when I returned to the text of the lecture, I could not fault Medawar. He is relentlessly honest that he is speculating and, in the context of the time, everything he suggests is eminently reasonable. I was also surprised at how much of the lecture is concerned with haemolytic disease of the newborn and antibodies, when it is so often quoted in the context of T-cell allorecognition (although T cells had not been discovered at the time). Why, then, is this piece so often used in this way?

Partly, it is because of the difficulties of getting hold of the text itself: if an illegal photocopy cannot be procured, the reader is compelled to make a trip to the British Library. But this is to ignore the question of why some of the ideas from this lecture have been passed from one reproductive immunologist to another, as an oral tradition. Surely, the answer is tied up with the legend of Medawar and from that our eagerness to claim him as the founder of our discipline, even when the man himself made the limitations of his thoughts on the matter clear.

## AUTHORS’ CONTRIBUTIONS 

V.M. contributed to the writing, reviewing, and editing of original draft.
